# Functional Limitations and Illness-Related Absenteeism among School-Aged Children with and without Long COVID, United States, 2022–2023

**DOI:** 10.3201/eid3114.251035

**Published:** 2025-12

**Authors:** Nicole D. Ford, Regina M. Simeone, Caroline Pratt, Sharon Saydah

**Affiliations:** Centers for Disease Control and Prevention, Atlanta, Georgia, USA

**Keywords:** COVID-19, respiratory infections, severe acute respiratory syndrome coronavirus 2, SARS-CoV-2, SARS, coronavirus disease, viruses, coronavirus, long COVID, disability, absenteeism, functional limitations, children, pediatric, United States

## Abstract

We examined functional limitations and illness-related chronic absenteeism (i.e., missing >18 days of school for health reasons) in a cross-sectional nationally representative sample of 11,057 US children 5–17 years of age who ever or never had long COVID (i.e., symptoms lasting >3 months after COVID-19 illness). Among 4,587 children with prior COVID-19, we estimated whether long COVID was associated with increased illness-related chronic absenteeism by using logistic regression. Our analysis showed that ≈1.4% of school-aged children had long COVID at some point. Among children with prior COVID-19, those who had long COVID at some point more frequently reported functional limitations, such as difficulty with memory, than those who did not have long COVID (18.3% vs. 8.6%). Having long COVID was associated with higher odds of illness-related chronic absenteeism. Children who had long COVID could experience functional limitations and absenteeism. School accommodations might be an option to improve functional limitations.

Long COVID is a chronic condition that includes a wide range of symptoms and conditions lasting >3 months after SARS-CoV-2 infection (*1*). Long COVID can affect multiple body systems, including cardiovascular, respiratory, and musculoskeletal. Commonly reported symptoms include fatigue, difficulty thinking or concentrating, and cough (*2*). In the 2023 US National Health Interview Survey (NHIS), 1.0% of children 6–11 years of age and 2.3% of children 12–17 years of age reported having long COVID at some point (*3*). Long COVID symptoms can limit a person’s ability to carry out day-to-day activities and affect functioning at school or work. In 2023, eight in 10 children with long COVID had activity limitation compared with before their COVID-19 illness (*3*).

Studies quantifying illness-related absenteeism in children by long COVID status are lacking, and few studies have examined functional limitations among children with long COVID. A qualitative study of UK children with long COVID reported that those children found attending school difficult, and even a gradual return required balancing the effects of missing school with preventing relapse (*4*). The larger societal effects of long COVID could be far-reaching if US school-aged children are unable to maintain school attendance, gain educational advancement, or engage in recreational activities vital to social and emotional development. We assessed whether functional limitations and illness-related absenteeism were more common among US school-aged children and adolescents who ever had long COVID compared with those who never had long COVID.

## Methods

We used data from the 2022 and 2023 NHIS, a large, nationally representative cross-sectional household survey of the civilian noninstitutionalized population of the United States (*5*,*6*). NHIS uses geographically clustered sampling techniques to select household units. One child 0–17 years of age is then sampled from each selected household. Interviewers collect information from the sample child’s parent or a knowledgeable adult during interviews. The sample child response rates for the overall surveys were 45.8% in 2022 and 44.9% in 2023 (*5*,*6*). We limited the analytic sample to school-aged children 5–17 years of age.

### Study Definition of Long COVID

Prior COVID-19 was determined by an affirmative response to NHIS survey questions about SARS-CoV-2 infection. In 2022, questions were “Has a doctor or other health professional ever told you that [NAME] had or likely had coronavirus or COVID-19?” and “Did [NAME] ever take a test that showed he or she had coronavirus or COVID-19?” and in 2023 “Has [NAME] ever had COVID-19?” In 2022, the survey ascertained ongoing COVID-19 symptoms among children with symptomatic mild, moderate, or severe COVID-19 and those who had unknown symptom severity. In 2023, the survey ascertained ongoing COVID-19 symptoms among all children with prior COVID-19 irrespective of symptom severity during acute illness. NHIS classified children as having long COVID when they had any symptoms lasting >3 months that they did not have before having COVID-19. NHIS classified children as never having long COVID if they never had COVID-19 or had COVID-19 at some point but never had ongoing symptoms.

### Functional Limitations

NHIS administers the Washington Group/UNICEF Child Functioning Module to identify the subpopulation of children who are at greater risk of experiencing limited participation in an unaccommodating environment (*7*). The 24-question module for children 5–17 years of age was designed and validated for use in censuses and population-based surveys (*7*). Functional domains of the module were vision, hearing, mobility, self-care, communication, learning, cognition, accepting change, behavior, relationships, and psychosocial. Because of the small number of children with vision, hearing, mobility, self-care, and communication limitations in the sample when stratified by long COVID status, we did not include those estimates because they did not meet National Center for Health Statistics (NCHS) data presentation standards on the basis of effective sample size, confidence interval width, number of events, and degrees of freedom (*8*). We classified indicators in the learning, cognition, accepting change, behavior, and relationships domains as binary yes/no variables where yes indicated children with any difficulty. In the psychosocial domain, we classified both the frequency of seeming very anxious, nervous, or worried and the frequency of seeming very sad or depressed as never, a few times per year, monthly, or weekly/daily.

### Illness-Related Absenteeism

Parents reported days of school the sample child missed because of illness or injury during the 12 months preceding the survey. We classified illness-related chronic absenteeism according to the US Department of Education’s definition of chronic absenteeism (*9*), which is missing >18 days of school (yes/no).

### Sociodemographic and Health Characteristics

Parents reported child-level sociodemographic characteristics including age (5–11 years or 12–17 years), sex (male or female), private health insurance (yes/no), and race and Hispanic ethnicity (Hispanic, non-Hispanic White, or other single or multiple races). Other single or multiple races included non-Hispanic Asian, non-Hispanic American Indian/Alaska Native, non-Hispanic Black, and other single and multiple races, which we aggregated to meet NCHS presentation standards because of small numbers. We included race and Hispanic ethnicity in this study to account for documented racial and ethnic differences in parent-reported long COVID in US children (*10*). Household-level characteristics included region (Northeast, Midwest, South, West), urban–rural classification (metropolitan, nonmetropolitan), and parental education (high school diploma or less, some college or associate’s degree, bachelor’s degree or higher). NCHS classifies region and urban–rural on the basis of household location (*11*).

Parent-reported child health characteristics were 12-month recall of COVID-19 vaccine receipt and use of prescription medication for emotions, concentration, behavior, or mental health (yes/no). NHIS did not collect information about prescription medication use for other conditions. Co-occurring conditions included chronic health conditions (i.e., asthma, prediabetes, diabetes) and neurodevelopmental conditions (i.e., attention-deficit/hyperactivity disorder [ADHD], autism, developmental delay, intellectual disability, and learning disability), coded yes/no.

### Statistical Analyses

We estimated the weighted prevalence (Clopper-Pearson 95% CI) of ever experiencing long COVID among the cohort. We then estimated weighted prevalences and 95% CIs of sociodemographic and health indicators, functional limitations, and sick days by long COVID status. For all indicators, we used Rao-Scott χ^2^ tests to examine differences by long COVID status.

During the COVID-19 pandemic, the Centers for Disease Control and Prevention published guidance on isolation following a positive SARS-CoV-2 test and quarantine following exposure (*12*). Because COVID-19 illness is associated with sick days, irrespective of whether long COVID develops, we created a restricted subsample of 4,587 children with prior COVID-19. That design helped control for confounding by acute COVID-19 effects, which might otherwise bias estimates in the full sample. We used logistic regression to estimate whether ever having long COVID was associated with increased occurrence of illness-related chronic absenteeism among children with prior COVID-19. We first ran an unadjusted model including only long COVID as the exposure. We then tested various adjusted models. We identified age, sex, race and Hispanic ethnicity, and parental education as potential confounders and assessed our study population by examining differences in unadjusted and adjusted estimates of association and model goodness of fit, favoring parsimony. We used Harrell’s C statistic to assess goodness of fit.

Because chronic health conditions and neurodevelopmental conditions can develop as the result of a SARS-CoV-2 infection, we did not consider those conditions for the primary models. However, those conditions potentially could confound the relationship between long COVID and illness-related chronic absenteeism. For example, diabetes might be associated with risk of developing long COVID and with missing days from school. To examine confounders, we conducted sensitivity analyses. First, we added chronic health conditions (any vs. none) to the final adjusted model, then we added neurodevelopmental conditions (any vs. none) to the model. We compared the estimates of association for long COVID in the final adjusted model to those in the models containing chronic health conditions and neurodevelopmental conditions.

We used SAS version 9.4 (SAS Institute, Inc., https://www.sas.com) to conduct analyses. We obtained weighted estimates by using SAS-callable SUDAAN (RTI International, https://www.rti.org), applying survey weights generated by NHIS (*5*,*6*) and accounting for complex sampling. We considered 2-sided p<0.05 statistically significant.

## Results

In total, NHIS surveyed 5,498 children 5–17 years of age in 2022 and 5,676 in 2023. We excluded 106 (0.9%) children missing information on prior COVID-19 and 11 (0.1%) missing information on ongoing symptoms following COVID-19. The final unweighted analytic sample included 11,057 children with a weighted value of 106,793,000.

On the basis of the weighted samples, we estimated 1,538,000 (1.4%) school-aged children had long COVID at some point (Table 1). Among those children, 69.7% were 12–17 years of age, 59.4% were female and 40.6% were male, and 35.2% were experiencing long COVID symptoms at time of survey. We found statistically significant differences by long COVID status for age group, sex, race and ethnicity, parental education, co-occurring chronic health conditions, neurodevelopmental conditions, learning disability, and use of prescription medication for emotions, concentration, behavior, or mental health.

**Table 1:** Sociodemographic and health characteristics of children in a study of functional limitations and illness-related chronic absenteeism among school-aged children with and without long COVID, United States, 2022–2023*

Characteristic	Weighted no. (%) [95% CI]		p value†	
	Had long COVID	Never had long COVID		
Total no.	1,538,000 (1.4) [1.2–1.7]	105,255,000 (98.6) [98.3–98.8]		
Age group, y			<0.001	
5–11	467,000 (30.3) [22.9–38.6]	55,319,000 (52.6) [51.5–53.6]		
12–17	1,072,000 (69.7) [61.4–77.1]	49,936,000 (47.4) [46.4–48.5]		
Sex			0.02	
M	625,000 (40.6) [32.1–49.5]	53,925,000 (51.3) [50.2–52.3]		
F	914,000 (59.4) [50.5–67.9]	51,274,000 (48.7) [47.7–49.8]		
Race and Hispanic ethnicity‡			0.002	
Hispanic	494,000 (32.1) [24.4–40.6]	27,188,000 (25.8) [24.0–27.7]		
Non-Hispanic White	874,000 (56.8) [48.2–65.1]	53,633,000 (51.0) [49.1–52.8]		
Another single or multiple races	171,000 (11.1) [6.6–17.3]	24,433,000 (23.2) [21.9–24.6]		
Private health insurance§	745,000 (48.4) [39.7–57.2]	58,410,000 (55.7) [54.2–57.2]	0.09	
Region			0.87	
Northeast	221,000 (14.4) [8.8–21.7]	16,382,000 (15.6) [14.3–16.9]		
Midwest	344,000 (22.4) [16.1–29.7]	21,691,000 (20.6) [19.2–22.1]		
South	571,000 (37.1) [29.1–45.7]	41,589,000 (39.5) [37.5–41.6]		
West	402,000 (26.1) [19.2–34.0]	25,593,000 (24.3) [22.5–26.3]		
Urban classification¶			0.35	
Metropolitan	1,289,000 (83.8) [76.2–89.8]	91,184,000 (86.6) [85.2–88.0]		
Non-metropolitan	249,000 (16.2) [10.2–23.8]	14,071,000 (13.4) [12.0–14.8]		
Parental education			<0.001	
High school diploma, GED, or less	367,000 (24.8) [17.8–32.9]	27,649,000 (27.0) [25.7–28.3]		
Some college or associate’s degree	576,000 (38.9) [30.5–47.8]	25,446,000 (24.8) [23.8–25.9]		
Bachelor’s degree or higher	538,000 (36.3) [28.1–45.1]	49,447,000 (48.2) [46.7–49.8]		
Received COVID-19 vaccine <12 mo	280,000 (18.2) [11.9–26.1]	22,232,000 (21.1) [20.1–22.2]	0.43	
Ever had COVID-19	1,538,000 (100.00) [NA]	41,511,000 (39.4) [38.3–40.6]	NA	
Current long COVID	542,000 (35.2) [27.1–44.1]	NA	NA	
Chronic health condition#	261,000 (17.0) [11.2–24.1]	9,131,000 (8.7) [8.1–9.3)	<0.001	
Neurodevelopmental condition**	432,000 (28.1) [20.5–36.7]	18,584,000 (17.7) [16.8–18.6]	0.002	
Intellectual disability	25,000 (1.7) [0.3–5.1]	1,581,000 (1.5) [1.2–1.8]	0.88	
Learning disability	225,000 (14.6) [8.8–22.3]	8,238,000 (7.8) [7.2–8.5]	0.007	
Prescription medication for emotions, concentration, behavior, or mental health <12 mo	382,000 (24.8) [17.6–33.3]	10,196,000 (9.7) [9.1–10.5]	<0.001	

*Data are from the National Health Interview Survey (*5*,*6*). Numbers are weighted, are rounded to the nearest thousand, and might vary because of missing data. All estimates are weighted, account for complex sampling, and meet National Center for Health Statistics data standards. Parents of children with prior COVID-19 (mild, moderate, or severe symptoms or unknown symptom severity during acute illness [2022] and all children with prior COVID-19 irrespective of acute illness symptom severity [2023]) were asked about ongoing symptoms using the question “Did [NAME] have any symptoms lasting 3 months or longer that [she/he] did not have prior to having COVID-19?” Children with an affirmative response were classified as having long COVID. Children who never had COVID-19 and those who had COVID-19 but never had ongoing symptoms were classified as never having long COVID. NA, not applicable. †p values are for Rao-Scott χ^2^ tests for sociodemographic and health characteristics comparing children who ever and never had long COVID. ‡Another single or multiple race(s) were non-Hispanic Asian, non-Hispanic American Indian/Alaska Native, non-Hispanic Black, and other single race and multiple races. §Private health insurance compared to Medicaid, other public coverage, and no insurance. ¶Urban–rural classification defined according to the 2013 National Center for Health Statistics Urban-Rural Classification Scheme for Counties (https://www.cdc.gov/nchs/data/series/sr_02/sr02_166.pdf). #Current asthma, prediabetes, or diabetes. **Current autism, attention-deficit hyperactivity disorder, developmental delay, intellectual disability, or learning disability.

Compared with children who never had long COVID, children who had long COVID had a higher prevalence of functional limitations across 5 of the 6 functional domains (Table 2). Within the cognition domain, compared with children who never had long COVID, children who ever had long COVID had approximately double the prevalence of difficulty with memory (18.3% vs. 8.6%) and difficulty concentrating (14.3% vs. 7.7%) (p<0.01 for both comparisons). Prevalence of learning difficulty was also roughly double among children who had long COVID at some point compared with those who had not (19.8% vs. 10.4%).

**Table 2:** Functional domains of children in a study of functional limitations and illness-related chronic absenteeism among school-aged children with and without long COVID, United States, 2022–2023*

Functional domain	Weighted no. (%) [95% CI]		p value†
	Had long COVID	Never had long COVID	
Learning difficulty	305,000 (19.8) [13.2–27.9]	10,929,000 (10.4) [9.7–11.1]	<0.001
Cognition			
Difficulty concentration	220,000 (14.3) [8.7–21.7]	8,120,000 (7.7) [7.1–8.3]	0.006
Difficulty remembering	282,000 (18.3) [11.6–26.7]	9,069,000 (8.6) [8.0–9.3]	<0.001
Behavior, difficulty controlling	282,000 (18.3) [12.1–26.0]	16,732,000 (15.9) [15.0–16.8]	0.45
Relationships, difficulty making friends	281,000 (18.4) [12.4–25.7]	11,852,000 (11.3) [10.6–12.0]	0.008
Difficulty accepting changes in routine	582,000 (37.8) [30.1–46.1]	24,173,000 (23.0) [22.0–24.0]	<0.001
Psychosocial			
Frequency of seeming anxious, nervous, or worried			<0.001
Never	437,000 (28.7) [21.4–36.8]	50,235,000 (47.8) [46.4–49.2]	
A few times per year	368,000 (24.2) [17.4–32.0]	25,437,000 (24.2) [23.2–25.3]	
Monthly	243,000 (15.9) [9.8–23.9]	10,994,000 (10.5) [9.8–11.2]	
Weekly or daily	477,000 (31.3) [23.6–39.8]	18,387,000 (17.5) [16.6–18.4]	
Frequency of seeming very sad or depressed			<0.001
Never	765,000 (49.8) [41.0–58.5]	70,865,000 (67.5) [66.2–68.7]	
A few times per year	286,000 (18.6) [12.8–25.7]	20,760,000 (19.8) [18.8–20.7]	
Monthly	195,000 (12.7) [7.7–19.4]	6,811,000 (6.5) [5.9–7.1]	
Weekly or daily	291,000 (18.9) [12.6–26.8]	6,552,000 (6.2) [5.7–6.8]	

*Data are from the National Health Interview Survey (*5*,*6*). Numbers are weighted, are rounded to the nearest thousand, and might vary because of missing data. All estimates are weighted, account for complex sampling, and meet National Center for Health Statistics data standards. Parents of children with prior COVID-19 (mild, moderate, or severe symptoms or unknown symptom severity during acute illness [2022] and all children with prior COVID-19 irrespective of acute illness symptom severity [2023]) were asked about ongoing symptoms using the question “Did [NAME] have any symptoms lasting 3 months or longer that [she/he] did not have prior to having COVID-19?” Children with an affirmative response were classified as having long COVID. Children who never had COVID-19 and those who had COVID-19 but never had ongoing symptoms were classified as never having long COVID. NA, not applicable. †p values are for Rao-Scott χ^2^ tests for sociodemographic and health characteristics comparing children who ever and never had long COVID.

In the relationship domain, children who had long COVID had a higher prevalence of difficulty making friends than children who never had long COVID (18.4% vs. 11.3%) (p = 0.008). Children who had long COVID also had more difficulty accepting changes in routine than children who never had long COVID (37.8% vs. 23.0%) (p<0.001). In the psychosocial domain, children who had experienced long COVID had higher prevalence of anxiety than children who never had long COVID (31.3% vs. 17.5% for weekly or daily anxiety) and for depression (18.9% vs. 6.2% for weekly or daily depression) (p<0.001 for both comparisons).

Among children who had experienced long COVID, 10.7% missed >30 days of school for health reasons during the year preceding the survey (Table 3). In both the full analytic sample and restricted subsample of children with prior COVID-19 illness, 13.9% of children who had experienced long COVID were chronically absent (i.e., missed >18 days). Among children who never had long COVID, 3.5% in the full analytic sample and 4.9% in the restricted subsample of children with prior COVID-19 were chronically absent for health reasons.

**Table 3:** Illness-related absenteeism among children in a study of functional limitations and illness-related absenteeism among school-aged children with and without long COVID, United States, 2022–2023*

Absenteeism	Weighted no. (%) [95% CI]		p value†
	Had long COVID	Never had long COVID	
Total analytic sample	1,538,000	105,255,000	
No. sick days			<0.001
0	311,000 (20.3) [13.5–28.5]	33,808,000 (32.4) [31.3–33.6]	
1–5	421,000 (27.4) [20.4–35.4]	47,782,000 (45.9) [44.8–47.0]	
6–17	588,000 (38.3) [29.7–47.6]	18,873,000 (18.1) [17.2–19.0]	
18–29	49,000 (3.2) [1.1–7.1]	2,203,000 (2.1) [1.8–2.5]	
>30	165,000 (10.7) [5.7–18.0]	1,530,000 (1.5) [1.2–1.8]	
Chronic absence‡	214,000 (13.9) [8.5–21.1]	3,733,000 (3.5) [3.1–4.0]	<0.001
Subsample of children with prior COVID-19	1,538,000	41,511,000	
No. sick days			<0.001
0	311,000 (20.3) [13.5–28.5]	9,473,000 (23.0) [21.6–24.6]	
1–5	421,000 (27.4) [20.4–35.4]	18,950,000 (46.1) [44.3–47.8]	
6–17	588,000 (38.3) [29.7–47.6]	10,659,000 (25.9) [24.4–27.5]	
18–29	49,000 (3.2) [1.1–7.1]	1,201,000 (2.9) [2.4–3.6]	
>30	165,000 (10.7) [5.7–18.0]	844,000 (2.1) [1.6–2.6]	
Chronic absence‡	214,000 (13.9) [8.5–21.1]	2,045,000 (4.9) [4.2–5.7]	<0.001

*Data are from the National Health Interview Survey (*5*,*6*). Numbers are weighted, are rounded to the nearest thousand, and might vary because of missing data. All estimates are weighted, account for complex sampling, and meet National Center for Health Statistics data standards. Parents of children with prior COVID-19 (mild, moderate, or severe symptoms or unknown symptom severity during acute illness [2022] and all children with prior COVID-19 irrespective of acute illness symptom severity [2023]) were asked about ongoing symptoms using the question “Did [NAME] have any symptoms lasting 3 months or longer that [she/he] did not have prior to having COVID-19?” Children with an affirmative response were classified as having long COVID. Children who never had COVID-19 and those who had COVID-19 but never had ongoing symptoms were classified as never having long COVID. NA, not applicable. †p values are for Rao-Scott χ^2^ tests for sociodemographic and health characteristics comparing children who ever and never had long COVID. ‡Chronic absence from school for health reasons was defined as missing >18 days of school due to illness or injury during the 12 months preceding the survey.

In the unadjusted model, long COVID was associated with 3.1 (95% CI 1.8–5.3; Harrell’s C statistic = 0.53) times the odds of illness-related chronic absenteeism (Figure; Appendix Table). In the adjusted multivariable model accounting for race and Hispanic ethnicity and parental education, having long COVID at some point was associated with 2.5 (95% CI 1.5–4.3; Harrell’s C statistic = 0.63) times the odds of illness-related chronic absenteeism compared with never having long COVID. In the sensitivity analyses, the effect sizes for long COVID were attenuated slightly after additionally controlling for chronic health conditions (adjusted odds ratio 2.4, 95% CI 1.4–4.2; Harrell’s C statistic = 0.64) and neurodevelopmental conditions (adjusted odds ratio 2.3, 95% CI 1.4–4.0; Harrell’s C statistic = 0.65) (Figure; Appendix Table).

**Figure:**
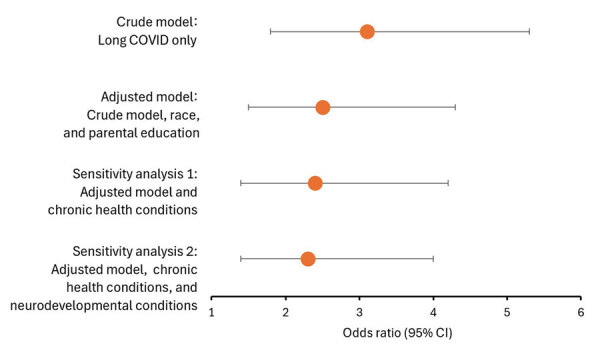
Adjusted odds ratios from a study of functional limitations and illness-related chronic absenteeism among school-aged children with and without long COVID, United States, 2022–2023. Graph shows adjusted odds ratios (dots) and 95% CIs (whiskers) for chronic absence from school for health reasons among children with prior COVID-19 illness comparing children who did and did not have long COVID. The study examined 4,587 school-aged children (5–17 years) who had COVID-19 illness identified through the National Health Interview Survey, 2022–2023 (*5*,*6*). Chronic absence from school for health reasons was defined as missing >18 days of school because of illness or injury (compared with 0–17 days) during the 12 months preceding the survey. The minimally adjusted model controls for race and Hispanic ethnicity and parental education. Chronic health conditions included asthma, prediabetes, and diabetes. Neurodevelopmental conditions include autism, attention-deficit hyperactivity disorder, intellectual disability, learning disability, and developmental delay.

## Discussion

In this nationally representative sample of US school-aged children during 2022–2023, 1.4% had experienced long COVID at some point, and long COVID disproportionately affected older and female children. We found approximately double the prevalence of functional limitations in the learning, cognition, relationships, accepting change, and psychosocial domains among children who had experienced long COVID compared with those who never had long COVID. In the absence of appropriate supports, those functional limitations could make academic achievement and engagement in social activities challenging. Nearly 14% of children who experienced long COVID were chronically absent from school for health reasons, and >1 in 10 missed >6 weeks during the 12 months preceding the survey. After controlling for sociodemographic and health characteristics, children who experienced long COVID had 2.3 times the adjusted odds of illness-related chronic absenteeism compared with those who never had long COVID.

Together, our findings suggest that long COVID has a potentially large impact on US school-aged children. Parents, caregivers, teachers, and schools may consider that children with long COVID disproportionately experience both functional limitations and sick days off from school. School accommodations, such as reduced workload and rest periods that are recommended for other conditions affecting cognitive and academic functioning, such as concussion or ADHD, could be options to improve outcomes (*13*,*14*).

Little information about functional limitation, sick days off, and long COVID in US children is available to put our study findings into context. Most of the available literature on long COVID in children was reported from medical records, convenience samples, and small case-based studies (*15*,*16*). However, studies of pediatric populations have described symptoms or conditions in long COVID, such as autonomic dysfunction (*17*), abnormalities in brain metabolism (*18*), and exercise intolerance (*19*). In 2023, 80.0% of children who had long COVID at the time of NHIS had >1 long COVID-associated activity limitation; however, the study did not examine specific functional limitations (*3*). Our findings are consistent with studies reporting positive associations between other chronic health conditions, such as type 1 diabetes and asthma, and school absence in children (*20*,*21*). For example, a study of asthma in urban US schools found that asthma explained 14%–18% of student absenteeism, after accounting for sociodemographic and health characteristics (*20*).

Cognitive, learning, relationship, accepting changes, and psychosocial functional limitations were roughly twice as common among children who had long COVID at some point compared with those who never had long COVID. In the absence of appropriate supports or accommodations, functional limitations might affect academic performance and social development. Neuropsychiatric symptoms, such as impairment to memory recall, executive dysfunction, and depression, are commonly reported postacute sequelae of SARS-CoV-2 infection (*22*,*23*). The prevalence of childhood mental, behavioral, and developmental disorders (MBDD) in the United States has increased broadly over time; data from the National Survey of Children’s Health showed MBDD prevalence among children 3–17 years of age increased from 25.3% to 27.7% during 2016–2021, with increases specific to learning disability, developmental delay, speech or language disorder, anxiety, and depression (*24*).

The association between COVID-19 and MBDD could be bidirectional. COVID-19 might influence MBDD prevalence indirectly through social determinants of health (e.g., social isolation), directly through infection (e.g., long COVID–associated cognitive impairment), or both (*25*). Conversely, chronic health, mental health, and neurodevelopmental conditions might be associated with long COVID because they increase the risk for SARS-CoV-2 infection, severe COVID-19 illness, and symptoms and conditions consistent with long COVID (*26*–*28*). For example, a study using 2022 National Survey on Health and Disability data found the prevalence of long COVID was higher among persons with preexisting disabilities compared with the general population (40.6% vs. 18.9%) (*29*). Furthermore, a 2023 systematic review and meta-analysis found that poor mental health increased the likelihood of developing long COVID in pediatric populations (*30*). Symptoms and conditions might develop, or underlying conditions might worsen after SARS-CoV-2 infection (*31*). Our study is cross-sectional and does not provide information regarding the onset of co-occurring conditions relative to the development of long COVID. Thus, our findings cannot be used to examine the direction of the effects. However, our findings highlight that children with long COVID could have complex needs.

Prescription medication use for emotions, concentration, behavior, or mental health were common among children in our study who had experienced long COVID; 1 in 4 reportedly used those medications during the 12 months preceding the survey. That finding might represent increased risk for long COVID among children with underlying psychological or neurodevelopmental health conditions or new onset long COVID–associated conditions requiring medication. Studies have reported increased use of those types of prescription medications since the pandemic, specifically in adults and school-aged girls. For example, a study of commercial healthcare claims found that the percentage of girls receiving stimulants, primarily those used to treat ADHD, increased by 8.3% for girls 10–15 years of age and 15.1% of those 15–19 years of age from 2020 to 2021 (*32*). In our study, long COVID was more prevalent in adolescents and girls. Similarly, the monthly rate of antidepressant dispensing to adolescents and young adults increased during 2016–2020, and the rate of change increased by 63.5% beginning March 2020 (*33*). Although those findings might reflect secular trends, studies have also shown differences in receipt of some medications by long COVID status. For example, adult patients with long COVID–associated fatigue and concentration problems were twice as likely to receive a stimulant prescription than patients with only acute COVID-19 illness, suggesting potential off-label use of stimulants to treat long COVID (*34*). Furthermore, stimulants can be prescribed to treat myalgic encephalomyelitis/chronic fatigue syndrome, a common postacute sequelae of SARS-CoV-2 infection (*35*,*36*).

Chronic absenteeism is a primary cause of poor academic achievement (*37*). Missing school makes keeping pace with schoolwork difficult, increases the likelihood of dropping out, and reduces opportunities to build relationships with peers (*37*). From 2018–19 to 2021–22 enrollment-weighted prevalence of chronic absenteeism in the United States increased from 14.8% to 28.3%, a 91% increase relative to the prepandemic timeframe (*38*). In our study, 1 in 7 children who experienced long COVID were chronically absent from school for health reasons, more than double the odds of children with only acute COVID-19 illness. That finding is consistent with literature on disability and chronic absenteeism in US children wherein children with disabilities more frequently experienced >15 missed days of school compared with children without disabilities (14.8% vs. 4.4%) (*39*). Moreover, having long COVID at any point was significantly associated with parental education level (p<0.001), and previous literature found that socioeconomic factors can affect the association of chronic health conditions and absenteeism (*40*). Schools might consider health-related factors in their ongoing efforts to improve school attendance (*41*) and could collaborate with healthcare systems to provide integrated systems of support to address complex needs for children with disabilities and health concerns (*42*).

Limited guidance exists to address long COVID–associated functional limitations or chronic absence resulting from long COVID in US school-aged children (*43*). Guidance related to return-to-work and reasonable accommodations for adults affected by long COVID (*44*) might not be applicable to children. The National Academies of Sciences, Engineering, and Medicine’s report on long COVID and disability highlight that long COVID could greatly affect disability and functioning in children and that long COVID is poorly understood in that population (*1*). Appropriate diagnosis and treatment of symptoms could improve functional limitations among children with long COVID. In addition, long COVID can be relapsing and remitting, so children might require flexible accommodations to meet changing needs. Furthermore, inadequate rest and pushing beyond functional limitations can worsen long COVID symptoms (*45*,*46*). Healthcare providers can collaborate with parents, caregivers, and schools to develop educational intervention and support for children with special educational needs (*47*).

Strengths of this study include a large, nationally representative sample of children with detailed information about sociodemographic and health characteristics. In addition, the tool used to measure functional limitations was specifically designed for population-based surveys and validated (*7*). 

The first limitation of this study was that long COVID and illness-related chronic absenteeism were based on parental report and could be subject to recall bias and misclassification. Second, identification of COVID-19 history varied slightly between survey years. In 2022, ongoing symptoms were not ascertained among children with asymptomatic COVID-19. In 2023, ongoing symptoms were ascertained among all children with prior COVID-19. Thus, children with asymptomatic COVID-19 or those with undetected COVID-19 might have been misclassified in the group without long COVID. In that case, our results might have been biased toward the null. Third, younger children have difficulty expressing ongoing symptoms, potentially leading to underreporting of long COVID. The Child Functioning Module was designed to be administered to caregivers in surveys so functional limitations could be less subject to misclassification. Fourth, missed days of school for health reasons was not specific to long COVID. Because acute COVID-19 illness is associated with missed time from school, we limited the modeling to the subsample of children who had prior COVID-19 illness to isolate the effect of long COVID on illness-related chronic absenteeism beyond acute illness. Fifth, the NHIS is cross sectional, so we did not have information about the timing of long COVID relative to chronic health or neurodevelopmental conditions, functional limitations, or absence from school. Long COVID–associated activity limitation was added to the 2023 survey but was not available for 2022. Finally, because of low prevalence of long COVID in children, stratified estimates for some chronic health and neurodevelopmental conditions did not comply with NCHS reporting standards. We combined response options for some sociodemographic characteristics to comply with reporting standards.

In summary, long COVID remains a public health concern in US school-aged children. Because children who had long COVID experienced a disproportionate burden of functional limitations compared with their peers, educational institutions need to recognize the potential for accommodations that support learning goals and social development. Because having long COVID at any point was strongly associated with illness-related chronic absenteeism among children with prior COVID-19, healthcare providers and schools could collaborate to recognize and support children experiencing long COVID to minimize effects on learning and development.

Appendix Additional information on functional limitations and illness-related chronic absenteeism among school-aged children with and without long COVID, United States, 2022–2023
