# Supplementary material for: Functional Limitations and Illness-Related Absenteeism among School-Aged Children with and without Long COVID, United States, 2022–2023
Source: Emerg Infect Dis. 2025 Dec;31(Suppl 2):S11–9. doi: 10.3201/eid3114.251035 (PMC12829551; doi:10.3201/eid3114.251035)
Supplement: Appendix — Additional information on functional limitations and illness-related chronic absenteeism among school-aged children with and without long COVID, United States, 2022–2023 [file 25-1035-Techapp-s1.pdf]

Article DOI: https://doi.org/10.3201/eid3114.251035

EID cannot ensure accessibility for supplementary materials supplied by authors. Readers who have difficulty accessing supplementary content should contact the authors for assistance.

Functional Limitations and Illness-Related Absenteeism among School-Aged Children with and without Long COVID, United States, 2022–2023

Appendix

Appendix Table 1. Crude and adjusted odds of illness-related chronic absenteeism among U.S. school aged children (5–17 y) with prior COVID-19 illness, National Health Interview Survey, 2022–2023*

Characteristic	Crude OR (95% CI)	Adjusted OR (95% CI)†	Sensitivity 1, aOR (95% CI)†	Sensitivity 2, aOR (95% CI)†
Exposure: Ever Long COVID (ref. never Long COVID)	3.1 (1.8, 5.3)	2.5 (1.5, 4.3)	2.4 (1.4, 4.2)	2.3 (1.4, 4.0)
Potential covariates				
Age (centered at 12 y)	1.0 (1.0, 1.1)	NA	NA	NA
Female sex (ref. male)	0.9 (0.7, 1.2)	NA	NA	NA
Hispanic (ref. other)	2.1 (1.3, 3.4)	1.3 (0.8, 2.1)	1.3 (0.8, 2.2)	1.3 (0.8, 2.3)
Non-Hispanic White (ref. other)	1.7 (1.1, 3.8)	2.4 (1.5, 3.7)	2.5 (1.6, 3.9)	2.3 (1.5, 3.7)
High school diploma or less (ref. BA degree or higher)	1.6 (1.1, 2.4)	1.9 (1.3, 2.9)	1.9 (1.4, 4.2)	1.8 (1.2, 2.7)
Some college (ref. BA degree or higher)	2.2 (1.5, 3.1)	2.3 (1.6, 3.3)	2.2 (1.2, 2.8)	2.1 (1.5, 3.7)
Chronic health condition(s)‡ (ref. no chronic health conditions)	2.1 (1.5, 3.1)	NA	2.0 (1.4, 3.0)	1.9 (1.3, 2.8)
Neurodevelopmental condition(s)§ (ref. no neurodevelopmental conditions)	2.4 (1.7, 3.3)	NA	NA	1.9 (1.3, 2.7)

*Data are from 4,587 children with prior COVID-19 illness and non-missing information about sick days. Chronic absence from school for health reasons was defined as missing 18 or more days of school due to illness or injury during the 12 mo preceding the survey (compared to 0–17 d). Parents of children with prior COVID-19 (mild, moderate, or severe symptoms or unknown symptom severity during acute illness [2022] and all children with prior COVID-19 irrespective of acute illness symptom severity [2023]) were asked about ongoing symptoms ("Did [child] have any symptoms lasting 3 months or longer that [she/he] did not have prior to having COVID-19?"). Those with an affirmative response were classified as ever experienced Long COVID. Goodness of fit for the models (Harrell's C statistic) are as follows: 0.53 for the model with only Long COVID; 0.63 for the adjusted model with Long COVID, race/ethnicity, and parental education; 0.64 for the model with Long COVID, race/ethnicity, parental education, and chronic health conditions; and 0.65 for the model with Long COVID, race/ethnicity, parental education, chronic health conditions, and neurodevelopmental conditions. aOR, adjusted odds ratio; NA, not applicable; OR, odds ratio.
†Adjusted ORs control for other indicators in the table.
‡Chronic health conditions include asthma, prediabetes, and diabetes.
§Neurodevelopmental conditions include autism, attention deficit hyperactivity disorder, intellectual disability, learning disability, and developmental delay.
